# Dichlorido(4,4′-di-*tert*-butyl-2,2′-bi­pyridine-κ^2^ *N*,*N*′)gold(III) tetrachlorido­aurate(III) acetonitrile solvate

**DOI:** 10.1107/S1600536808025646

**Published:** 2008-08-20

**Authors:** Sema Öztürk Yıldırım, Mehmet Akkurt, Nasser Safari, Vahid Amani, Vickie McKee, Anita Abedi, Hamid Reza Khavasi

**Affiliations:** a Department of Physics, Faculty of Arts and Sciences, Erciyes University, 38039 Kayseri, Turkey; b Chemistry Department, Shahid Beheshti University, Evin, Tehran 1983963113, Iran; c Department of Chemistry, Loughborough University, Leicestershire LE11 3TU, England; d Department of Chemistry, North Tehran Branch, Islamic Azad University, Tehran, Iran

## Abstract

In the title compound, [AuCl_2_(C_9_H_12_N)_2_][AuCl_4_]·C_2_H_3_N, there is a mirror plane passing through Au and the central C—C bond of the bipyridyl ligand in the cation, and through Au and two Cl atoms of the anion. A *cis*-AuCl_2_N_2_ square-planar geometry for the cation and a square-planar AuCl_4_ geometry for the anion result. The two C atoms and the N atom of the acetonitrile mol­ecule all have *m* site symmetries. In the crystal structure, weak C—H⋯Cl inter­actions may help to establish the packing.

## Related literature

For related structures, see: Abbate *et al.* (2000 ▶); Adams & Strähle (1982 ▶); Bjernemose *et al.* (2004 ▶); Hayoun *et al.* (2006 ▶); McInnes *et al.* (1995 ▶).
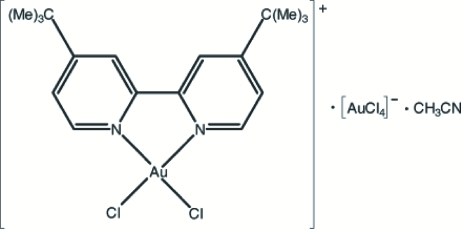


## Experimental

### 

#### Crystal data

[AuCl_2_(C_9_H_12_N)_2_][AuCl_4_]·C_2_H_3_N
*M* *_r_* = 916.09
Monoclinic, 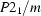

*a* = 6.7880 (9) Å
*b* = 14.2270 (19) Å
*c* = 14.1330 (19) Å
β = 97.151 (2)°
*V* = 1354.3 (3) Å^3^
*Z* = 2
Mo *K*α radiation
μ = 11.43 mm^−1^
*T* = 150 (2) K
0.14 × 0.10 × 0.01 mm

#### Data collection

Bruker APEXII CCD diffractometer
Absorption correction: multi-scan (*SADABS*; Sheldrick, 2003 ▶) *T*_min_ = 0.298, *T*_max_ = 0.894
14949 measured reflections
3888 independent reflections
2860 reflections with *I* > 2σ(*I*)
*R*_int_ = 0.060

#### Refinement

*R*[*F* ^2^ > 2σ(*F* ^2^)] = 0.036
*wR*(*F* ^2^) = 0.079
*S* = 1.01
3888 reflections
155 parameters
H-atom parameters constrained
Δρ_max_ = 1.59 e Å^−3^
Δρ_min_ = −1.24 e Å^−3^

### 

Data collection: *APEX2* (Bruker, 2005 ▶); cell refinement: *APEX2*; data reduction: *SAINT* (Bruker, 2005 ▶); program(s) used to solve structure: *SIR92* (Altomare *et al.*, 1993 ▶); program(s) used to refine structure: *SHELXL97* (Sheldrick, 2008 ▶); molecular graphics: *ORTEP-3* (Farrugia, 1997 ▶); software used to prepare material for publication: *WinGX* (Farrugia, 1999 ▶).

## Supplementary Material

Crystal structure: contains datablocks global, I. DOI: 10.1107/S1600536808025646/hb2776sup1.cif

Structure factors: contains datablocks I. DOI: 10.1107/S1600536808025646/hb2776Isup2.hkl

Additional supplementary materials:  crystallographic information; 3D view; checkCIF report

## Figures and Tables

**Table:** 

Au1—Cl1	2.2590 (17)
Au1—N1	2.020 (4)
Au2—Cl2	2.271 (2)
Au2—Cl3	2.2675 (16)
Au2—Cl4	2.311 (2)

**Table:** 

N2—C11—C10	179.5 (14)

**Table 2:** Hydrogen-bond geometry (Å, °)

*D*—H⋯*A*	*D*—H	H⋯*A*	*D*⋯*A*	*D*—H⋯*A*
C3—H3⋯Cl3^i^	0.93	2.66	3.561 (6)	162
C3—H3⋯Cl1^ii^	0.93	2.64	3.231 (6)	122

Symmetry codes: (i) 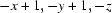; (ii) 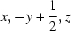.

**Fig. 1.**
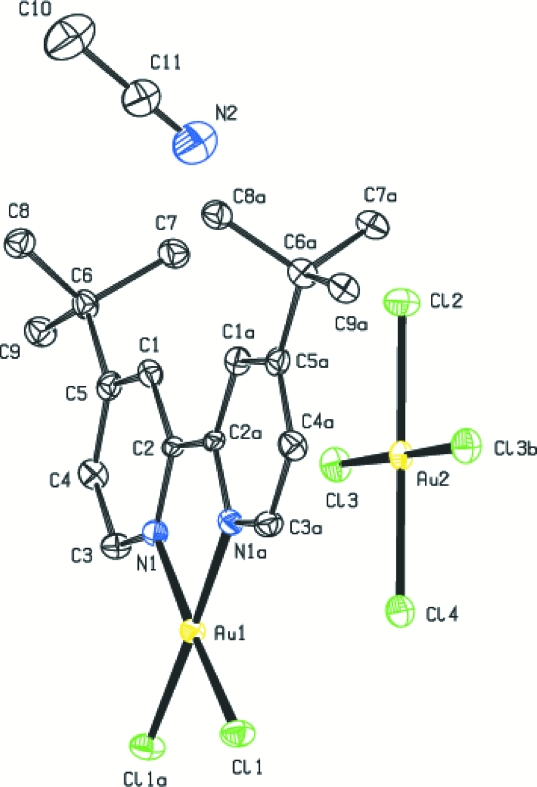
View of the molecular structure of (I) with displacement ellipsoids for non-H atoms drawn at the 30% probability level and H atoms omitted for clarity. The symmetry codes a and b both refer to (x, 1/2 - y, z).

**Fig. 2.**
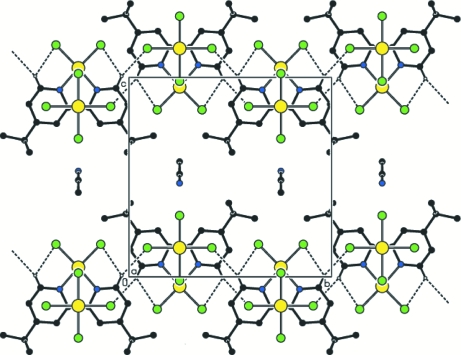
A view of the packing and the hydrogen bonding (dashed lines) of (I) down the a axis in the unit cell.

**Fig. 3.**
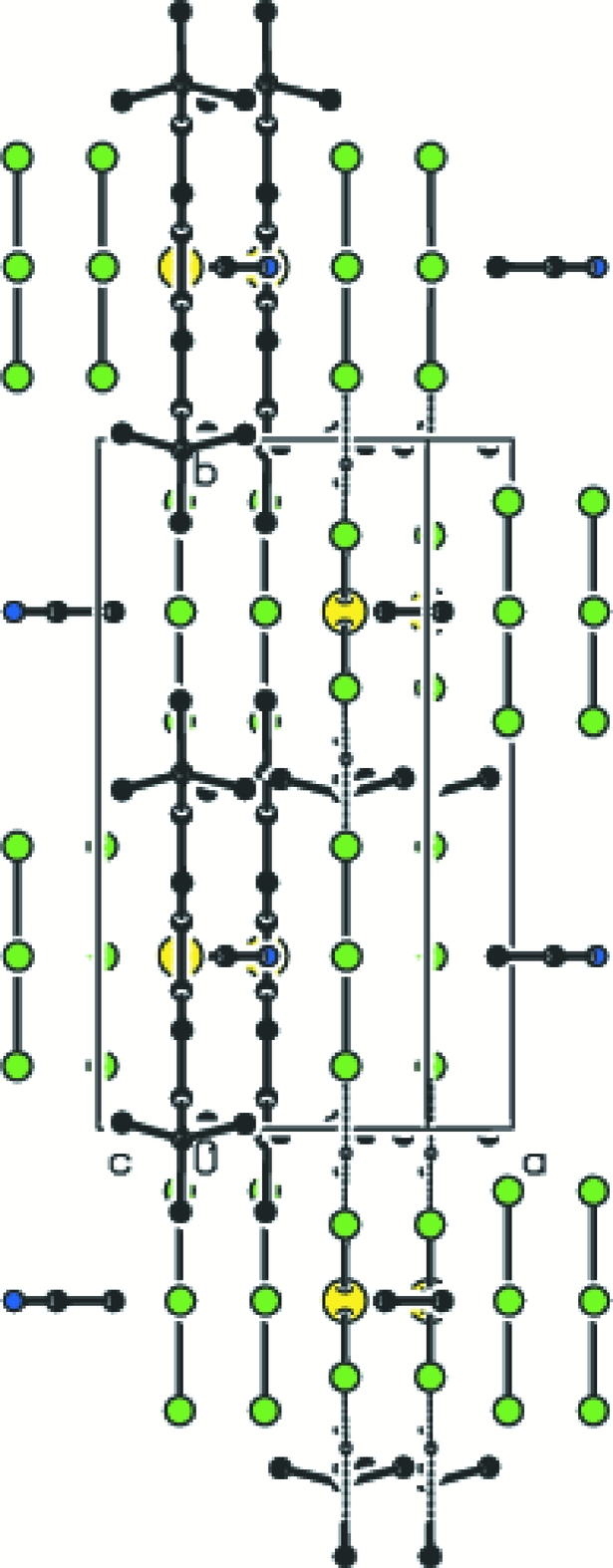
View of the unit-cell packing of (I) down the c axis.

## supplementary crystallographic information

### Comment

Several Au^III^ complexes, with formula, [AuCl_2_(N—N)], such as [AuCl_2_(bipy)][BF_4_], (II), (McInnes *et al.*, 1995), [AuCl_2_(bipy)](NO_3_), (III), (Bjernemose *et al.*, 2004), [AuCl_2_(bipy)][AuBr_4_], (IV), (Hayoun *et al.*, 2006) and [AuCl_2_(phen)]Cl.H_2_O, (V), (Abbate *et al.*, 2000) [where bipy is 2,2'-bipyridine and phen is 1,10-phenanthroline] have been synthesized and characterized by single-crystal X-ray diffraction methods.

Other Au^III^ complexes, with formula, [AuCl_2_*L*_2_], such as [AuCl_2_(py)_2_][AuCl_4_], (VI) and [AuCl_2_(py)_2_]Cl.H_2_O, (VII), (Adams & Strähle 1982) [where py is pyridine] have also bee prepared and characterized. We report herein the synthesis and crystal structure of the title compound, (I).

The asymmetric unit of (I) (Fig. 1) contains one half-cation, one half-anion and one half-acetonitrile molecule; the whole assemblage is symmetric according to a mirror plane. Both Au ions have square-planar coordination (Table 1) and the individual bond lengths and angles are in good agreement with the corresponding values in (II), (III), (IV), (V), (VI) and (VII).

In the crystal of (I), weak intermolecular C—H···Cl hydrogen bonds (Table 2) link the molecules to form a supramolecular structure (Fig. 2 and Fig. 3).

### Experimental

A solution of 4,4'-di-*tert*-butyl-2,2'-bipyridine (0.15 g, 0.56 mmol) in acetonitrile (40 ml) was added to a solution of HAuCl_4_.3H_2_O, (0.22 g, 0.56 mmol) in EtOH (50 ml) and the resulting yellow solution was stirred for 10 min at 313 K. Then, it was left to evaporate slowly at room temperature. After one week, yellow laths and prisms of (I) were isolated (yield 0.38 g; 74.0%).

### Refinement

All H atoms were positioned geometrically (C—H = 0.93-0.96Å) and refined as riding with *U*_iso_(H) = 1.2*U*_eq_(C) or 1.5*U*_eq_(methyl C).

### Crystal data

**Table:**

[AuCl_2_(C_9_H_12_N_1_)_2_][AuCl_4_]·C_2_H_3_N	*F*_000_ = 856
*M**_r_* = 916.09	*D*_x_ = 2.247 Mg m^−^^3^
Monoclinic, *P*2_1_/*m*	Mo *K*α radiation λ = 0.71069 Å
Hall symbol: -P 2yb	Cell parameters from 2450 reflections
*a* = 6.7880 (9) Å	θ = 2.9–24.8º
*b* = 14.2270 (19) Å	µ = 11.43 mm^−^^1^
*c* = 14.1330 (19) Å	*T* = 150 (2) K
β = 97.151 (2)º	Lath, yellow
*V* = 1354.3 (3) Å^3^	0.14 × 0.10 × 0.01 mm
*Z* = 2	

### Data collection

**Table:**

Bruker APEXII CCD diffractometer	3888 independent reflections
Radiation source: sealed tube	2860 reflections with *I* > 2σ(*I*)
Monochromator: graphite	*R*_int_ = 0.060
*T* = 150(2) K	θ_max_ = 29.5º
φ and ω scans	θ_min_ = 2.0º
Absorption correction: multi-scan(SADABS; Sheldrick, 2003)	*h* = −9→9
*T*_min_ = 0.298, *T*_max_ = 0.894	*k* = −19→19
14949 measured reflections	*l* = −19→18

### Refinement

**Table:**

Refinement on *F*^2^	Secondary atom site location: difference Fourier map
Least-squares matrix: full	Hydrogen site location: inferred from neighbouring sites
*R*[*F*^2^ > 2σ(*F*^2^)] = 0.036	H-atom parameters constrained
*wR*(*F*^2^) = 0.079	*w* = 1/[σ^2^(*F*_o_^2^) + (0.0318*P*)^2^] where *P* = (*F*_o_^2^ + 2*F*_c_^2^)/3
*S* = 1.01	(Δ/σ)_max_ < 0.001
3888 reflections	Δρ_max_ = 1.60 e Å^−^^3^
155 parameters	Δρ_min_ = −1.24 e Å^−^^3^
Primary atom site location: structure-invariant direct methods	Extinction correction: none

### Special details

**Table:**

Geometry. Bond distances, angles *etc*. have been calculated using the rounded fractional coordinates. All su's are estimated from the variances of the (full) variance-covariance matrix. The cell e.s.d.'s are taken into account in the estimation of distances, angles and torsion angles
Refinement. Refinement on *F*^2^ for ALL reflections except those flagged by the user for potential systematic errors. Weighted *R*-factors *wR* and all goodnesses of fit *S* are based on *F*^2^, conventional *R*-factors *R* are based on *F*, with *F* set to zero for negative *F*^2^. The observed criterion of *F*^2^ > σ(*F*^2^) is used only for calculating -*R*-factor-obs *etc*. and is not relevant to the choice of reflections for refinement. *R*-factors based on *F*^2^ are statistically about twice as large as those based on *F*, and *R*-factors based on ALL data will be even larger.

### Fractional atomic coordinates and isotropic or equivalent isotropic displacement parameters (Å^2^)

**Table:**

	*x*	*y*	*z*	*U*_iso_*/*U*_eq_	Occ. (<1)
Au1	0.23789 (4)	0.25000	−0.04727 (2)	0.0241 (1)	
Cl1	0.2075 (2)	0.13933 (12)	−0.16278 (11)	0.0366 (5)	
N1	0.2670 (6)	0.3421 (3)	0.0624 (3)	0.0238 (14)	
C1	0.3143 (8)	0.3571 (4)	0.2320 (4)	0.0258 (17)	
C2	0.2920 (7)	0.3000 (4)	0.1509 (4)	0.0222 (16)	
C3	0.2636 (8)	0.4365 (4)	0.0544 (4)	0.0286 (17)	
C4	0.2861 (8)	0.4940 (4)	0.1338 (4)	0.0303 (17)	
C5	0.3113 (8)	0.4559 (4)	0.2252 (4)	0.0277 (17)	
C6	0.3361 (8)	0.5158 (4)	0.3150 (4)	0.0287 (17)	
C7	0.5416 (9)	0.4930 (4)	0.3701 (4)	0.0345 (19)	
C8	0.1694 (9)	0.4913 (4)	0.3758 (4)	0.0333 (19)	
C9	0.3254 (9)	0.6207 (4)	0.2926 (5)	0.035 (2)	
N2	0.3784 (17)	0.25000	0.4696 (8)	0.066 (4)	
C10	0.104 (2)	0.25000	0.5775 (11)	0.087 (6)	
C11	0.2608 (17)	0.25000	0.5153 (9)	0.049 (4)	
Au2	0.79109 (4)	0.25000	0.14539 (2)	0.0258 (1)	
Cl2	0.8353 (4)	0.25000	0.30734 (16)	0.0402 (8)	
Cl3	0.7908 (2)	0.40938 (11)	0.14566 (12)	0.0363 (5)	
Cl4	0.7455 (3)	0.25000	−0.01937 (17)	0.0364 (7)	
H1	0.33140	0.32920	0.29200	0.0310*	
H3	0.24570	0.46360	−0.00600	0.0340*	
H4	0.28430	0.55890	0.12610	0.0360*	
H7A	0.64220	0.50110	0.32870	0.0520*	
H7B	0.54290	0.42920	0.39220	0.0520*	
H7C	0.56740	0.53460	0.42370	0.0520*	
H8A	0.18300	0.52950	0.43220	0.0500*	
H8B	0.17890	0.42610	0.39360	0.0500*	
H8C	0.04260	0.50290	0.33950	0.0500*	
H9A	0.43180	0.63750	0.25710	0.0520*	
H9B	0.33720	0.65570	0.35110	0.0520*	
H9C	0.20060	0.63480	0.25560	0.0520*	
H10A	0.07320	0.18640	0.59320	0.1300*	0.500
H10B	−0.01270	0.27970	0.54530	0.1300*	0.500
H10C	0.14820	0.28390	0.63500	0.1300*	0.500

### Atomic displacement parameters (Å^2^)

**Table:**

	*U*^11^	*U*^22^	*U*^33^	*U*^12^	*U*^13^	*U*^23^
Au1	0.0226 (2)	0.0279 (2)	0.0216 (2)	0.0000	0.0016 (1)	0.0000
Cl1	0.0483 (9)	0.0351 (8)	0.0257 (8)	0.0000 (7)	0.0023 (7)	−0.0050 (7)
N1	0.023 (2)	0.024 (2)	0.024 (3)	−0.0009 (19)	0.0014 (19)	−0.003 (2)
C1	0.023 (3)	0.025 (3)	0.029 (3)	0.002 (2)	0.002 (2)	0.004 (2)
C2	0.014 (2)	0.038 (3)	0.015 (3)	0.000 (2)	0.003 (2)	0.001 (2)
C3	0.033 (3)	0.029 (3)	0.024 (3)	−0.001 (3)	0.004 (2)	0.002 (3)
C4	0.032 (3)	0.025 (3)	0.033 (3)	−0.001 (2)	0.000 (3)	0.000 (3)
C5	0.021 (3)	0.032 (3)	0.031 (3)	−0.001 (2)	0.007 (2)	−0.001 (3)
C6	0.029 (3)	0.024 (3)	0.032 (3)	0.005 (2)	0.000 (3)	−0.002 (3)
C7	0.036 (3)	0.036 (4)	0.030 (3)	0.002 (3)	−0.002 (3)	−0.006 (3)
C8	0.034 (3)	0.035 (4)	0.031 (3)	−0.002 (3)	0.004 (3)	−0.007 (3)
C9	0.036 (3)	0.035 (4)	0.032 (4)	−0.001 (3)	−0.001 (3)	−0.003 (3)
N2	0.081 (8)	0.053 (6)	0.068 (7)	0.0000	0.020 (6)	0.0000
C10	0.109 (12)	0.079 (10)	0.080 (10)	0.0000	0.043 (9)	0.0000
C11	0.061 (7)	0.035 (6)	0.051 (7)	0.0000	0.012 (6)	0.0000
Au2	0.0194 (2)	0.0261 (2)	0.0318 (2)	0.0000	0.0033 (1)	0.0000
Cl2	0.0509 (14)	0.0399 (13)	0.0290 (12)	0.0000	0.0018 (10)	0.0000
Cl3	0.0365 (8)	0.0275 (8)	0.0448 (10)	−0.0009 (6)	0.0046 (7)	0.0040 (7)
Cl4	0.0272 (10)	0.0439 (13)	0.0377 (12)	0.0000	0.0022 (9)	0.0000

### Geometric parameters (Å, °)

**Table:**

Au1—Cl1	2.2590 (17)	C1—H1	0.9300
Au1—N1	2.020 (4)	C3—H3	0.9300
Au1—Cl1^i^	2.2590 (17)	C4—H4	0.9300
Au1—N1^i^	2.020 (4)	C7—H7B	0.9600
Au2—Cl2	2.271 (2)	C7—H7A	0.9600
Au2—Cl3	2.2675 (16)	C7—H7C	0.9600
Au2—Cl4	2.311 (2)	C8—H8A	0.9600
Au2—Cl3^i^	2.2675 (16)	C8—H8C	0.9600
N1—C3	1.348 (7)	C8—H8B	0.9600
N1—C2	1.378 (7)	C9—H9B	0.9600
N2—C11	1.088 (17)	C9—H9C	0.9600
C1—C5	1.409 (8)	C9—H9A	0.9600
C1—C2	1.398 (8)	C10—C11	1.462 (19)
C2—C2^i^	1.423 (8)	C10—H10B^i^	0.9600
C3—C4	1.382 (8)	C10—H10C^i^	0.9600
C4—C5	1.392 (8)	C10—H10A^i^	0.9600
C5—C6	1.521 (8)	C10—H10A	0.9600
C6—C8	1.544 (8)	C10—H10B	0.9600
C6—C9	1.526 (8)	C10—H10C	0.9600
C6—C7	1.545 (8)		
			
Cl1—Au1—N1	176.24 (13)	H7A—C7—H7C	109.00
Cl1—Au1—Cl1^i^	88.38 (6)	C6—C7—H7C	109.00
Cl1—Au1—N1^i^	95.38 (13)	H7A—C7—H7B	109.00
Cl1^i^—Au1—N1	95.38 (13)	H7B—C7—H7C	109.00
N1—Au1—N1^i^	80.86 (17)	C6—C8—H8C	110.00
Cl1^i^—Au1—N1^i^	176.24 (13)	C6—C8—H8A	109.00
Cl2—Au2—Cl3^i^	89.91 (4)	C6—C8—H8B	109.00
Cl2—Au2—Cl3	89.91 (4)	H8A—C8—H8B	109.00
Cl2—Au2—Cl4	179.90 (8)	H8A—C8—H8C	109.00
Cl3^i^—Au2—Cl4	90.10 (4)	H8B—C8—H8C	109.00
Cl3—Au2—Cl4	90.10 (4)	H9A—C9—H9B	109.00
Cl3—Au2—Cl3^i^	179.77 (6)	H9A—C9—H9C	110.00
Au1—N1—C2	113.8 (3)	H9B—C9—H9C	110.00
Au1—N1—C3	125.7 (4)	C6—C9—H9A	109.00
C2—N1—C3	120.5 (5)	C6—C9—H9B	109.00
C2—C1—C5	121.7 (5)	C6—C9—H9C	109.00
C1—C2—C2^i^	125.5 (5)	N2—C11—C10	179.5 (14)
N1—C2—C2^i^	115.8 (5)	C11—C10—H10C^i^	110.00
N1—C2—C1	118.7 (5)	C11—C10—H10A	110.00
N1—C3—C4	121.5 (5)	C11—C10—H10B	110.00
C3—C4—C5	120.8 (5)	C11—C10—H10C	110.00
C1—C5—C6	120.2 (5)	C11—C10—H10A^i^	110.00
C1—C5—C4	116.8 (5)	C11—C10—H10B^i^	110.00
C4—C5—C6	123.0 (5)	H10A^i^—C10—H10B	60.00
C8—C6—C9	108.5 (5)	H10B—C10—H10B^i^	52.00
C5—C6—C7	107.6 (4)	H10B—C10—H10C^i^	141.00
C5—C6—C8	109.0 (5)	H10A^i^—C10—H10C	52.00
C5—C6—C9	112.2 (5)	H10B^i^—C10—H10C	141.00
C7—C6—C8	110.5 (5)	H10C—C10—H10C^i^	60.00
C7—C6—C9	109.1 (5)	H10A^i^—C10—H10B^i^	109.00
C5—C1—H1	119.00	H10A^i^—C10—H10C^i^	109.00
C2—C1—H1	119.00	H10B^i^—C10—H10C^i^	109.00
N1—C3—H3	119.00	H10A—C10—H10B	109.00
C4—C3—H3	119.00	H10A—C10—H10C	109.00
C5—C4—H4	120.00	H10A—C10—H10A^i^	141.00
C3—C4—H4	120.00	H10A—C10—H10B^i^	60.00
C6—C7—H7B	109.00	H10A—C10—H10C^i^	52.00
C6—C7—H7A	109.00	H10B—C10—H10C	109.00
			
Cl1^i^—Au1—N1—C2	−179.5 (3)	N1—C2—C2^i^—N1^i^	0.0 (6)
Cl1^i^—Au1—N1—C3	0.5 (4)	N1—C2—C2^i^—C1^i^	−179.8 (5)
N1^i^—Au1—N1—C2	0.5 (3)	C1—C2—C2^i^—N1^i^	179.8 (5)
N1^i^—Au1—N1—C3	−179.6 (4)	C1—C2—C2^i^—C1^i^	0.0 (8)
Au1—N1—C2—C1	179.8 (4)	N1—C3—C4—C5	−0.6 (8)
Au1—N1—C2—C2^i^	−0.4 (5)	C3—C4—C5—C1	0.6 (8)
C3—N1—C2—C1	−0.2 (7)	C3—C4—C5—C6	−179.8 (5)
C3—N1—C2—C2^i^	179.6 (5)	C1—C5—C6—C7	60.9 (6)
Au1—N1—C3—C4	−179.6 (4)	C1—C5—C6—C8	−58.9 (7)
C2—N1—C3—C4	0.4 (8)	C1—C5—C6—C9	−179.1 (5)
C5—C1—C2—N1	0.2 (8)	C4—C5—C6—C7	−118.7 (6)
C5—C1—C2—C2^i^	−179.6 (5)	C4—C5—C6—C8	121.4 (6)
C2—C1—C5—C4	−0.3 (8)	C4—C5—C6—C9	1.3 (8)
C2—C1—C5—C6	−180.0 (5)		

Symmetry codes: (i) *x*, −*y*+1/2, *z*.

### Hydrogen-bond geometry (Å, °)

**Table:**

*D*—H···*A*	*D*—H	H···*A*	*D*···*A*	*D*—H···*A*
C3—H3···Cl3^ii^	0.93	2.66	3.561 (6)	162
C3—H3···Cl1^i^	0.93	2.64	3.231 (6)	122

Symmetry codes: (ii) −*x*+1, −*y*+1, −*z*; (i) *x*, −*y*+1/2, *z*.
